# Efficacy and safety of angiogenesis inhibitors in advanced gastric cancer: a systematic review and meta-analysis

**DOI:** 10.1186/s13045-016-0340-8

**Published:** 2016-10-18

**Authors:** Jing Yu, Yue Zhang, Lai-Han Leung, Lian Liu, Fan Yang, Xiaojun Yao

**Affiliations:** 1 Department of Oncology, Beijing Friendship Hospital, Capital Medical University, No. 95 Yong An Road, Xicheng District, Beijing, 100050 China; 2 State Key Laboratory of Quality Research in Chinese Medicine, Macau Institute for Applied Research in Medicine and Health, Macau University of Science and Technology, Taipa, Macau 999078 China

**Keywords:** Angiogenesis, Gastric cancer, Monoclonal antibodies, Tyrosine kinase inhibitors

## Abstract

Monoclonal antibodies and small molecule tyrosine kinase inhibitors (TKIs) directed against the vascular endothelial growth factor (VEGF) or its receptors have been investigated in several studies for the treatment of advanced gastric cancer (GC). In the present study, we aimed to evaluate the efficacy and safety of angiogenesis inhibitors in advanced GC. We searched published randomized controlled trials (RCTs) comparing angiogenesis inhibitors with non-angiogenesis inhibitors for the treatment of GC. MEDLINE, EMBASE, and the Cochrane Controlled Trials Register were searched. The extracted data on progression-free survival (PFS) and overall survival (OS) were measured in terms of hazard ratios (HR) and corresponding 95 % confidence intervals (CIs). In addition, risk ratios (RR) and corresponding 95 % CIs were pooled for objective response rate (ORR), disease control rate (DCR), and risk of adverse events (AEs). Ten RCTs involving 2786 patients were included. Compared with non-angiogenesis inhibitor-containing regimens, angiogenesis inhibitor-containing regimens resulted in a significant improvement in OS (HR 0.80, 95 % CI 0.69–0.93, *P* = 0.004), prolonged PFS (HR 0.66, 95 % CI 0.51–0.86, *P* = 0.002), and superior ORR (RR 1.34, 95 % CI 1.09–1.65, *P* = 0.005) and DCR (RR 1.37, 95 % CI 1.17–1.61, *P* = 0.0001). Angiogenesis inhibitors were associated with a greater number of AEs, but most of these were predictable and manageable. However, hand-foot syndrome, diarrhea, and gastrointestinal (GI) perforation were significantly increased in patients treated with angiogenesis inhibitors. In summary, angiogenesis inhibitor-containing regimens were superior to non-angiogenesis inhibitor-containing regimens in terms of OS, PFS, RR, and DCR in patients with advanced GC.

**Electronic supplementary material:**

The online version of this article (doi:10.1186/s13045-016-0340-8) contains supplementary material, which is available to authorized users.

## Background

Gastric cancer (GC) is the fifth most common malignancy worldwide and is the third leading cause of cancer deaths in both sexes, accounting for 723,000 deaths (8.8 % of the total), with the highest estimated mortality rates in East Asia and the lowest in North America [1–3]. Despite a significant decline in incidence worldwide over the last few decades, unfortunately, most GC patients are diagnosed at an advanced stage with 5-year overall survival (OS) rates for all stages combined generally below 30 %. Palliative systemic chemotherapy usually represented by a platinum-based doublet is the mainstay of treatment in advanced stages. The addition of a third drug such as an anthracycline or a taxane has been shown to improve response rate and provide modest survival benefits at the cost of significant toxicity. Progress in understanding GC cancer biology has led to the development of treatment targeting the epidermal growth factor receptor (EGFR), human epidermal growth factor receptor-2 (HER-2), and angiogenesis, which has changed the therapeutic paradigm of GC.

Recent studies have shown that angiogenesis in GC is a key step in metastasis. It has been confirmed that the vascular endothelial growth factor (VEGF) family is a crucial mediator of angiogenesis [4]. Approximately 50 % of GCs express VEGF, and the overexpression of VEGF-A and VEGF-D in GC is associated with a poor prognosis [4, 5]. Two categories of agents have been developed to target this family: antibody-based agents and VEGF receptor (VEGFR) tyrosine kinase inhibitors (TKIs) [6–8].

Many clinical trials have demonstrated that GC patients can benefit from angiogenesis inhibitors [9–14]. Ramucirumab (a type of monoclonal antibody) or apatinib (a type of TKI), which binds to VEGFR-2, are reported to increase progression-free survival (PFS) and OS in patients treated with one or two previous lines of therapy [9–11, 13]. However, many phase I/II studies of anti-angiogenic TKIs do not show satisfactory outcomes when added to chemotherapy [15–21]. The major studies of angiogenesis inhibitors for gastric cancer were showed in Additional file 1: Table S5. Therefore, the overall efficacy and safety of anti-angiogenic agents in GC are still unknown. In this study, we performed an updated meta-analysis to summarize the efficacy and safety of angiogenesis inhibitors in patients with advanced GC.

## Materials and methods

### Search strategy

**Fig. 1:**
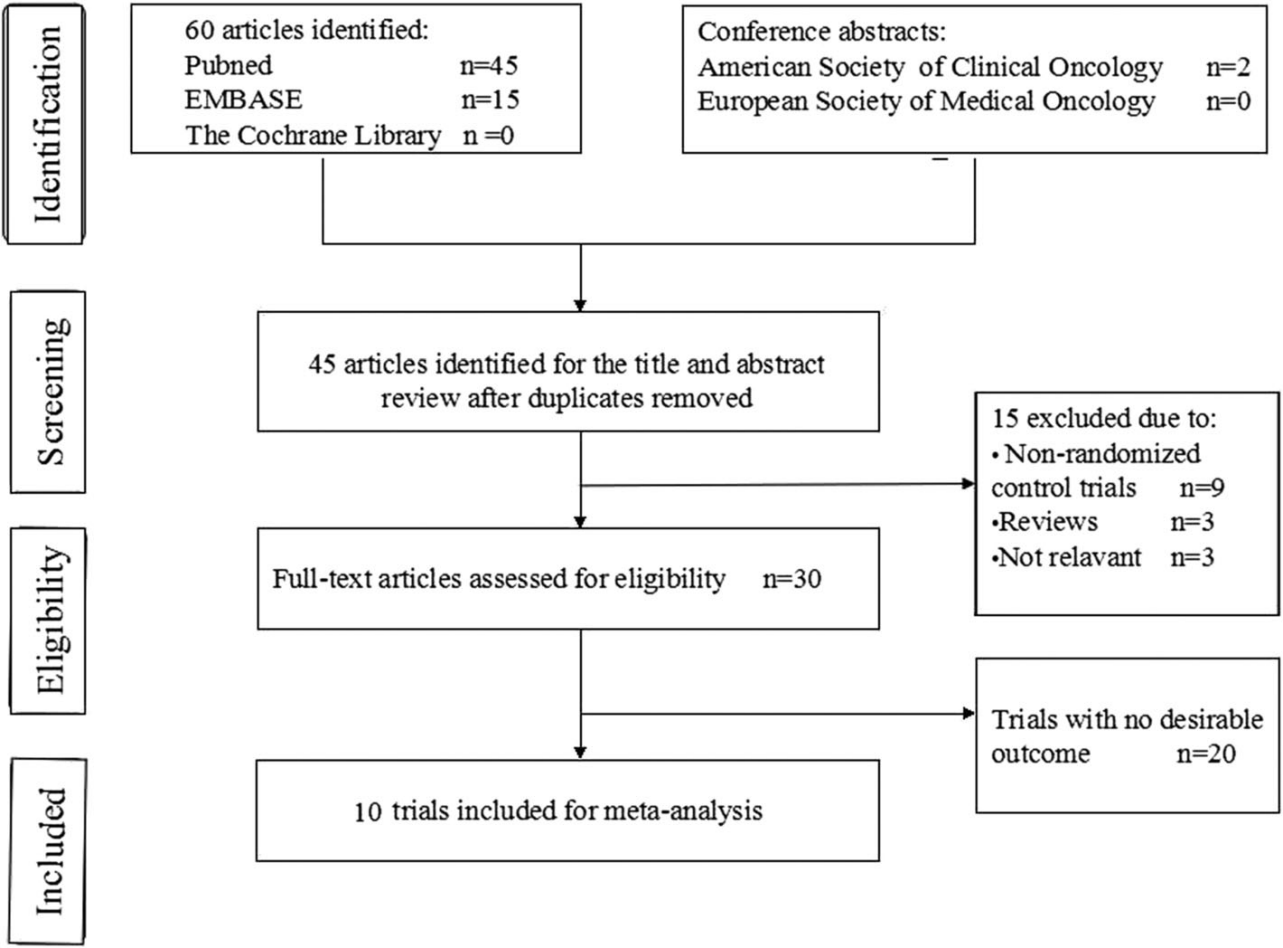
Flow chart of search process

An electronic search of the PubMed, MEDLINE, Cochrane Central Register of Controlled Trials (CENTRAL), and EMBASE databases as well as the American Society of Clinical Oncology (ASCO) and the European Society of Medical Oncology (ESMO) databases was performed from inception to February 2016. The detailed search strategy is described in Fig. 1. The search strategy included a combination of the MeSH term “angiogenesis inhibitors” OR the keywords “angiogenetic inhibitors,” “angiogenic antagonists,” “angiogenic inhibitors,” “angiostatic agents,” “antiangiogenetic agents,” “angiogenesis factor inhibitor”; the MeSH term “gastric neoplasms” OR the keywords “gastric tumor*,” “gastric neoplasm*,” “gastric cancer*”. All potentially relevant studies were retrieved, and their references were checked for additional eligible studies. Furthermore, we also searched http://www.clinicaltrials.gov/ for information on registered RCTs to identify trials registered as completed but whose results had not yet been published. This review was conducted and reported according to the Preferred Reporting Items for Systematic Reviews and Meta-Analysis (PRISMA) Statement issued in 2009.

### Definition of angiogenesis inhibitors

We defined angiogenesis inhibitors as those drugs which targeted VEGF and its receptors, which are the key mediators of angiogenesis.

### Inclusion criteria

Studies which met the following criteria were included: (1) patients must be cytologically or pathologically confirmed as having GC at a clinically advanced stage; (2) randomized controlled trials (RCTs) comparing angiogenesis inhibitors with non-angiogenesis inhibitors were deemed eligible; (3) one or more of the following were reported in the trials: overall response rate (ORR) (the sum of complete [CR] and partial responses [PR]), disease control rate (DCR) (the sum of CR, PR and stable disease [SD]), PFS, OS, and adverse events.

### Data extraction

Two independent investigators extracted data from the included studies on the basis of PRISMA [22]. When the two investigators disagreed, a third investigator participated in the discussion to resolve the disagreement. Information collected from these trials included the first author, year of publication, study design, number of patients, median age, ECOG performance status, therapeutic regimen, drug doses, and outcomes. Clinical outcomes collected from the trials included median PFS and OS, hazard ratios (HR) for OS and PFS and their 95 % confidence intervals (CIs), DCR and ORR, risk ratios (RR) for DCR and ORR, and their 95 % CIs, numbers, and rate of each type of adverse event stratified by severity. The response was evaluated according to the Response Evaluation Criteria in Solid Tumors (RECIST, version 1.1) and classified as a CR, PR, SD, or progressive disease (PD). ORR was defined as CR plus PR and DCR was defined as ORR plus SD.

### Quality assessment

The risk of bias in each study was assessed using the Cochrane Collaboration tool. The following evaluation domains were assessed accordingly: randomization sequence generation, allocation concealment, blinding of participants and study personnel, blinding of outcome assessors, incomplete outcome data, selective reporting, and other biases. The risk of each domain was rated as high risk, unclear risk, or low risk according to the match level between information extracted and evaluation criteria.

### Statistical analysis

A statistical analysis was conducted, and forest plots were performed using Review Manager 5.3. RRs and their 95 % CIs were calculated for DCR, ORR, and grade 3 and 4 toxicity as dichotomous outcomes. HRs were summarized and their corresponding standard errors were computed to analyze the time-to-event data as generic inverse variance outcomes. The inverse variance algorithm and Mantel-Haenszel algorithm were used. Heterogeneity between studies was assessed with Cochrane’s *X* ^2^ statistics and the inconsistency statistic (*I* ^2^). We considered *I* ^2^ < 50 % as low-level heterogeneity and *I* ^2^ > 50 % as significant heterogeneity. A fixed-effect model was used when *I* ^2^ < 50 % and a random-effect model was used when *I* ^2^ > 50 %. *P* values <0.05 were regarded as statistically significant in all included studies.

## Results

### Characteristics of the included studies

**Fig. 2:**
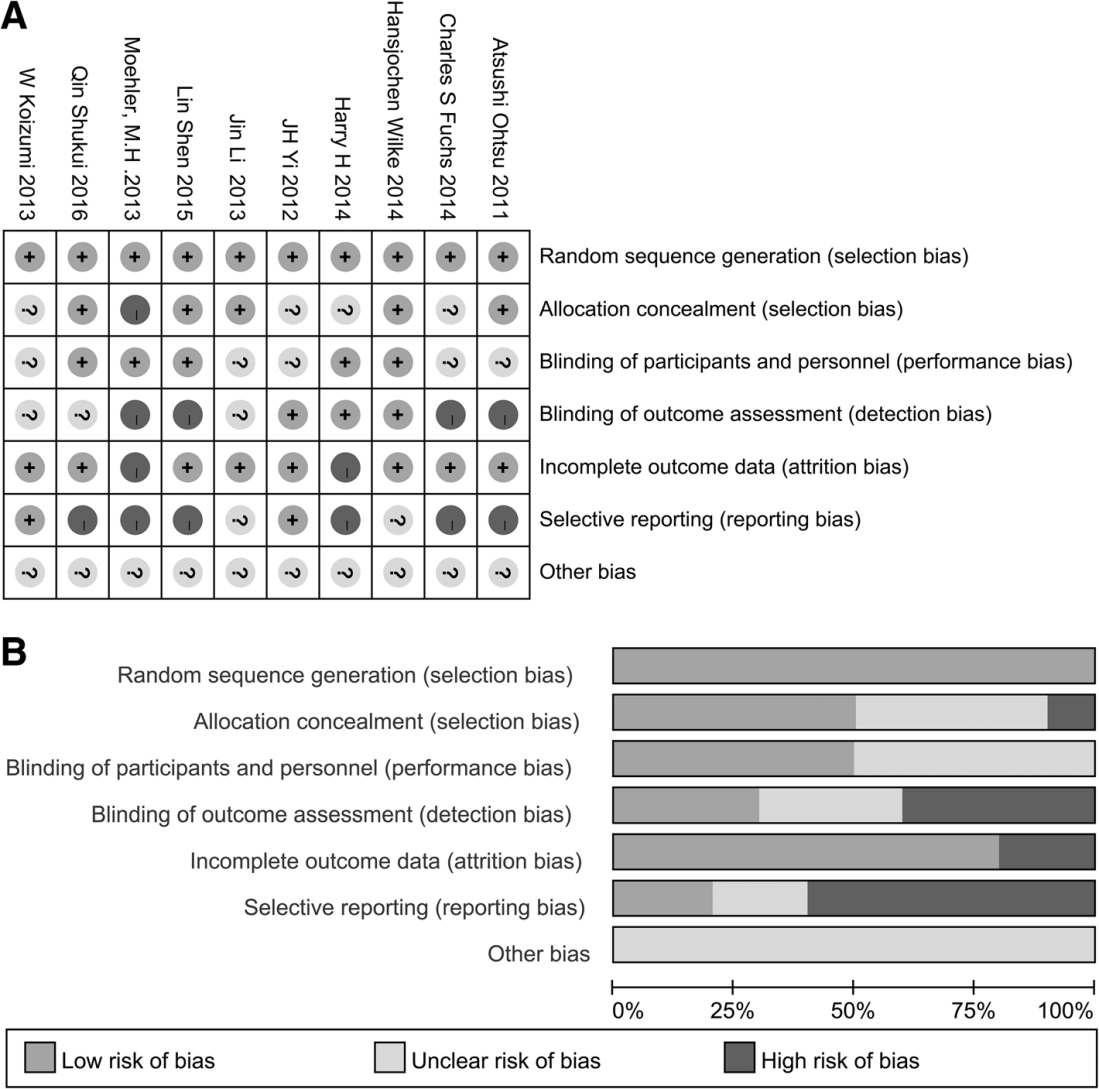
Assessment of risk of bias. **a** Risk of bias summary. **b** Risk of bias graph

Figure 1 shows the flow chart of study selection. A total of 60 relevant studies were identified by comprehensive search, and two conference abstracts were obtained by manual searching of the ASCO. Fifteen articles were excluded as they were duplicates, leaving 45 articles potentially eligible for inclusion, of which 15 were eliminated after reading the abstracts and titles. The full texts of the remaining 30 articles were then reviewed, and ten trials [9–11, 13, 18, 23–27] involving 2786 patients were finally included in the meta-analysis. The sample size in the included trials varied from 91 to 774, the median age of the enrolled patients ranged from 52 to 65 years. Of these, two studies [25, 26] enrolled patients who were treated with anti-VEGF-based drugs, five studies [9–11, 13, 27] enrolled patients who were treated with anti-VEGFR-2 agents, and three trials [18, 23, 24] enrolled patients who were treated with inhibitors of multiple tyrosine kinases (one of the targets is VEGFR-2). Four trials [18, 25–27] were conducted in the first-line setting and the other six trials [9–11, 13, 23, 24] in the pretreatment setting. Table 1 and Fig. 2 summarize the characteristics and qualities of both the included agents and articles.

**Table 1:** Characteristics of the included studies

Study	Agents	Year	Phase	Line	Regimens	No. of patients	Median age (years)	mOS (months)	mPFS (months)	DCR (%)	ORR (%)
Charles S. Fuchs	Ram	2014	3	2	Ram + BSC	238	60	5.20	2.10	49	3
					Placebo + BSC	117	60	3.80	1.30	23	3
										*P* = 0.76	*P* < 0.0001
Hansjochen Wilke	Ram	2014	3	2	Ram + PTX	330	61	9.60	4.40	80	28
					Placebo + PTX	335	61	7.40	2.90	64	16
										*P* < 0.0001	*P* = 0.0001
Harry H.	Ram	2014	2	1	Ram + mFOLFOX6	84	65	6.40	11.70	85	45
					Placebo + mFOLFOX6	84	60	6.70	11.50	67	46
										*P* = 0.008	
Jin Li	Apatinib	2013	2	>2	Apatinib	47	55	4.83	3.67	7.32	6.38
					Apatinib	46	53	4.27	3.20	15	13.04
					Placebo	48	54	2.50	1.40	0	0
Shukui Qin	Apatinib	2016	3	>2	Apatinib	176	58	6.50	2.60	42.05	2.84
					Placebo	91	58	4.70	1.80	8.79	0
										*P* = 0.1695	*P* < 0.001
Atsushi Ohtsu	Bev	2011	3	1	Bev + CDP + Cap	387	58	12.10	6.70	76.9	46
					Placebo + CDP + Cap	387	59	10.10	5.30	67.7	37.4
											*P* = 0.0315
Lin Shen	Bev	2015	3	1	Bev + CDP + Cap	100	54.2	10.50	6.30	75.3	41
					Placebo + CDP + Cap	102	55.5	11.40	6.00	72.1	34
											P = 0.35
Markus Hermann Moehler	Sunitinib	2013	2	2	Sunitinib + FOLFIRI	45	NR	10.50	3.60	58	20
					Placebo + FOLFIRI	46	NR	9.00	3.30	56	29
JH Yi	Sunitinib	2012	2	2	Sunitinib + docetaxel	56	54	8.00	3.90 (TTP)	75	41.1
					Docetaxel	49	52	6.60	2.60 (TTP)	51	14.3
W Koizumi	TSU-68	2013	2	1	TSU-68 + S-1/CDDP	45	62	16.6	6.9	NR	62.2
					S-1/CDDP	46	63.5	15.45	7.1	NR	56.5

*Ram* Ramucirumab, *BSC* best supportive care, *mOS* median overall survival, *mPFS* median progression-free survival, *HR* hazard ratio, *DCR* disease control rate, *ORR* objective response rate, *PTX* paclitaxel, *Bev* bevacizumab, *CDP/CDDP* cisplatin, *Cap* capecitabine, *TTP* time to progression, *NR* no report

### Assessment of methodological quality

We critically appraised the methodological quality of the included studies in accordance with the Cochrane Collaboration Risk of Bias Tool. All included trials were rated as low bias risk in randomization, as the authors stated the principles of randomization in detail. Other bias sources were not identified. The graphical results of methodological quality are shown in Fig. 2.

### Overall survival (OS) and progression-free survival (PFS)

**Fig. 3:**
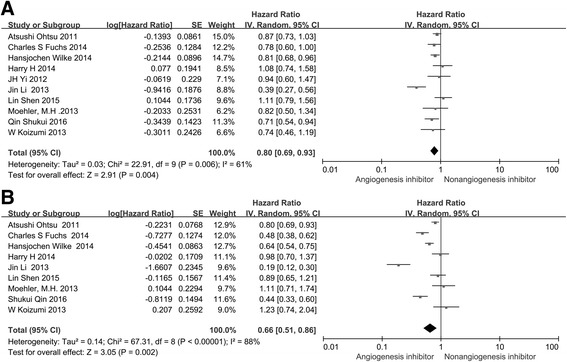
Forest plot and pooled HR and 95 % CI for OS (**a**) and PFS (**b**): anti-angiogenesis therapy versus non-anti-angiogenesis therapy. The pooled HR for OS and PFS showed that the patients receiving anti-angiogenesis therapy possessed a significant improvement in OS and PFS. *HR* hazard ratios, *OS* overall survival, *PFS* progression-free survival, *CI* confidence intervals

All included studies [9–11, 13, 18, 23–27] reported OS, and nine trials [9–11, 13, 18, 24–27] reported PFS. One study [23] reported time to progression (TTP). Of the ten trials, four [9–11, 13] reported a statistically significant improvement in OS and five trials [9–11, 13, 25] showed improved PFS. The median OS in the angiogenesis inhibitor groups reported in ten trials ranged from 4.27 to 12.1 months, and the median PFS varied from 2.1 to 9.6 months. The pooled results showed that when compared to the non-angiogenesis inhibitor groups, treatment with angiogenesis inhibitors were associated with a significantly prolonged OS (HR 0.80, 95 % CI 0.69–0.93, *P* = 0.004 Fig. 3a) and increased PFS (HR 0.66, 95 % CI 0.51–0.86, *P* = 0.002, Fig. 3b). Significant heterogeneity was detected among the studies in Fig. 3a (*P* = 0.006, *I* ^2^ = 61 %) and Fig. 3b (*P* < 0.00001, *I* ^2^ = 88 %), so we conducted a sensitivity analysis. We excluded the study of Atsushi Ohtsu that had the maximum relative weight (about 15.0 %) in Fig. 3a, the study of Moehler HM which had the minimum relative weight (about 6.2 %) in Fig. 3a, the study of Atsushi Ohtsu that had the maximum relative weight (about 12.9 %) in Fig. 3b, and the study of W Koizumi which had the minimum relative weight (about 9.0 %) in Fig. 3b, and the survival outcome was similar.

**Fig. 4:**
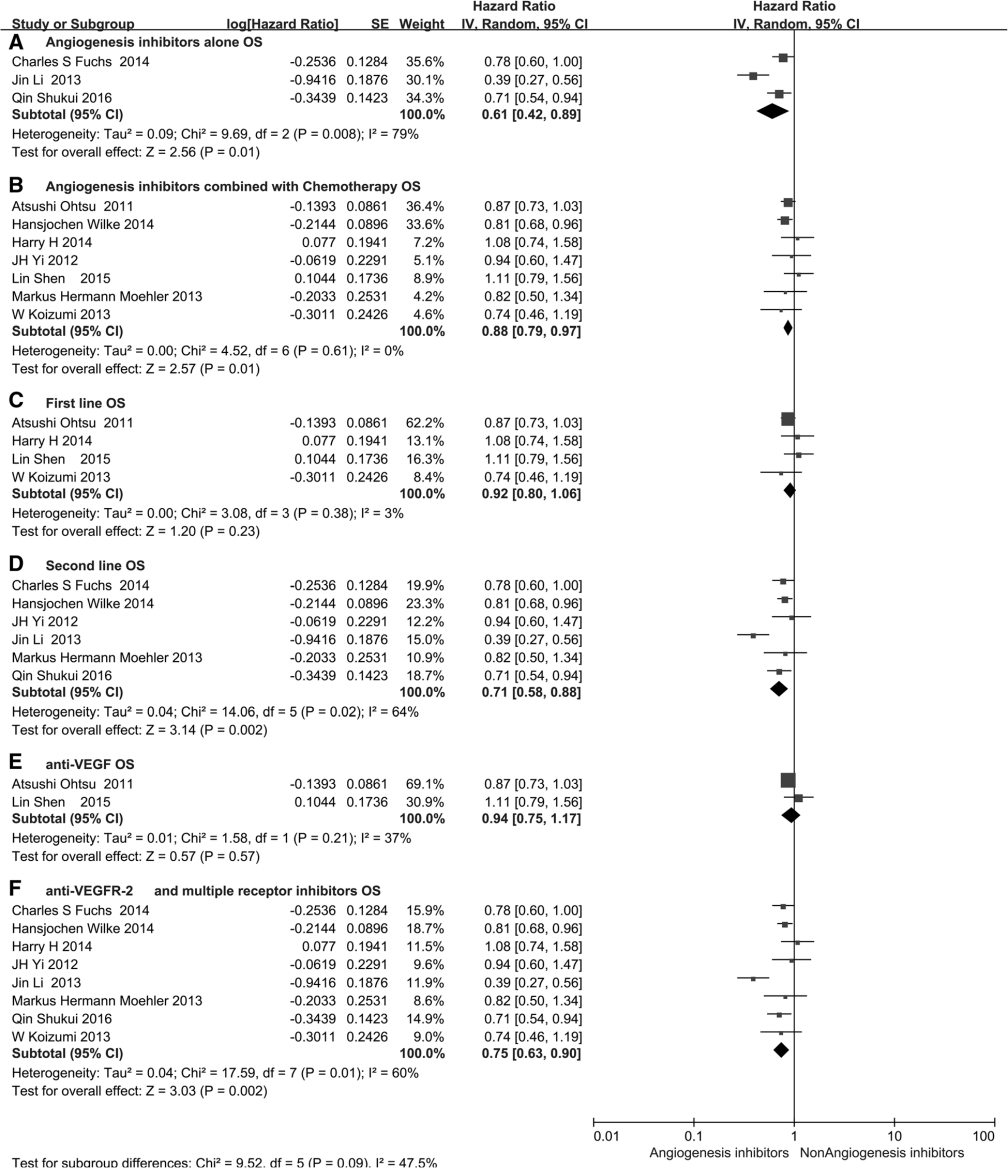
Forest plot and pooled HR and 95% CI for subgroup OS: anti-angiogenesis therapy versus non-anti-angiogenesis therapy. *HR* hazard ratios, *CI* confidence intervals, *OS* overall survival. (﻿﻿﻿**a**﻿: ﻿O﻿S of subgroups of angiogenesis inhibitors alone threapy; **b**: ﻿O﻿S of subgroups of angiogenesis inhibitors combined with chemotherapy threapy; **c**:﻿ O﻿S﻿ ﻿of subgr﻿oups ﻿of the first line﻿ ﻿thre﻿apy; **d**: OS of subgroups of the second line threapy; **e**: ﻿O﻿S of subgroups of anti-VEGF threapy; **f**:﻿ O﻿S of subgroups of anti-VEGFR an﻿d multiple receptor inhibitors threapy)﻿﻿

**Fig. 5:**
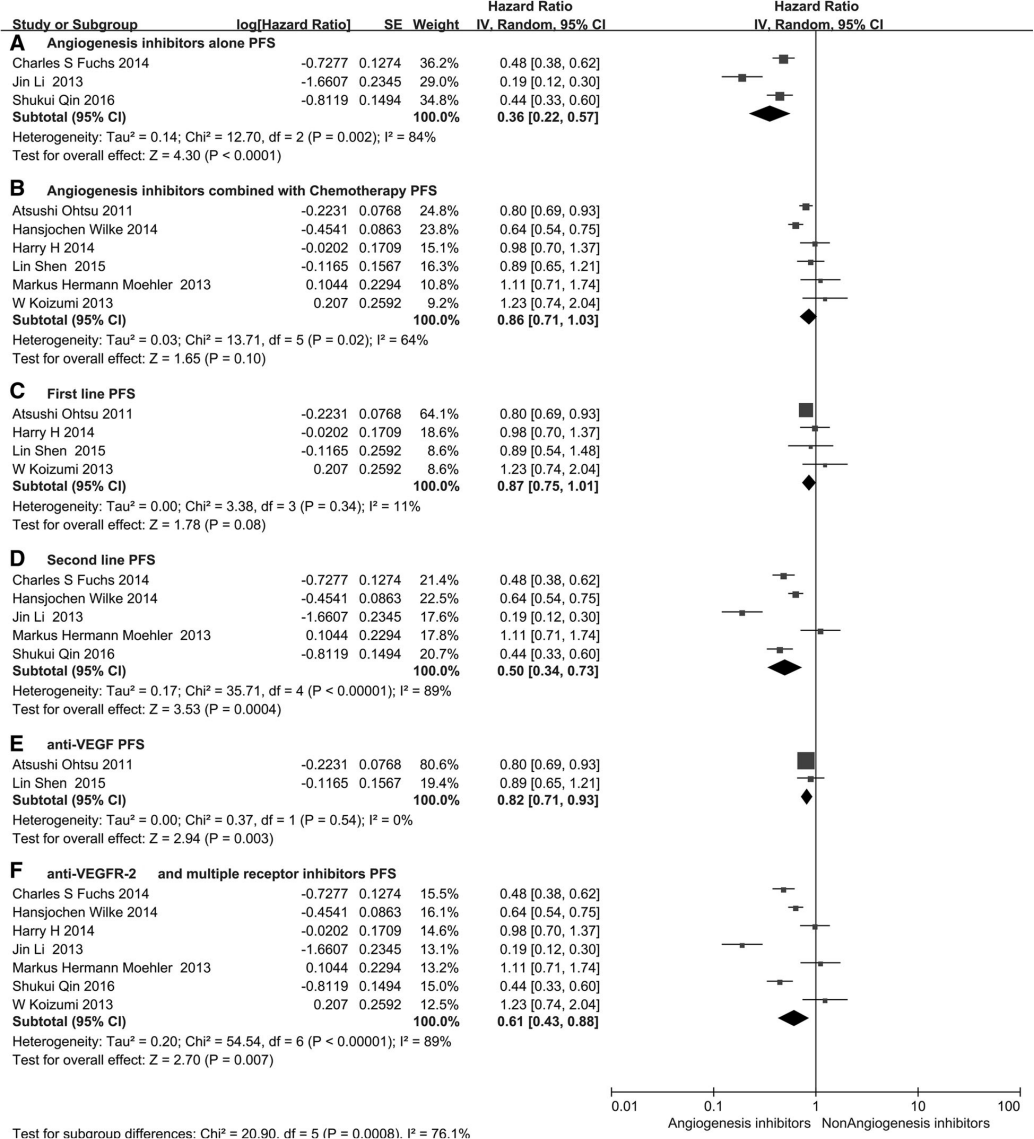
Forest plot and pooled HR and 95 % CI for subgroup PFS: anti-angiogenesis therapy versus non-anti-angiogenesis therapy. *HR* hazard ratios, *CI* confidence intervals, *PFS* progression-free survival. (﻿﻿**a**: PF﻿S of subgroups of a﻿ngiogenesis inhibitors alone threapy; **b**: ﻿PFS of subgroups of angiogenesis inhibitors combined with chemotherapy threapy; **c**:﻿ PFS of subgroups of the first line﻿ ﻿thre﻿apy; **d**: PFS of subgroups of the second line threapy; **e**:﻿ PFS of subgroups of anti-VEGF threapy; **f**: ﻿PFS of subgroups﻿ ﻿of anti-VEGFR an﻿d multiple receptor inhibitors threapy﻿﻿)﻿﻿

In the subgroup analyses of medication administered, both treatment with angiogenesis inhibitors alone (HR 0.61, 95 % CI 0.42–0.89, *P* = 0.01, Fig. 4a) and angiogenesis inhibitors combined with chemotherapy (HR 0.88, 95 % CI 0.79–0.97, *P* = 0.01, Fig. 4b) led to significantly improved OS, but only the treatment with angiogenesis inhibitors alone prolonged PFS (HR 0.36, 95 % CI 0.22–0.57, *P* < 0.0001, Fig. 5a). With regard to the line of treatment, a significant PFS (HR 0.50, 95 % CI 0.34–0.73, *P* = 0.0004, Fig. 5d) and OS (HR 0.71, 95 % CI 0.58–0.88, *P* = 0.002, Fig. 4d) benefit was found in ≥ the second-line treatment. Disappointingly, no PFS (HR 0.87, 95 % CI 0.75–1.01, *P* = 0.08, Fig. 5c) and OS (HR 0.92, 95 % CI 0.80–1.06, *P* = 0.23, Fig. 4c) benefits were observed with the first-line treatment. When stratified by drug class, anti-VEGF-based agents led to longer PFS (HR 0.82, 95 % CI 0.71–0.93, *P* = 0.003, Fig. 5e) but did not significantly improve OS (HR 0.94, 95 % CI 0.75–1.17, *P* = 0.57, Fig. 4e) compared with non-angiogenesis inhibitors. Anti-VEGFR-2 and multiple receptor inhibitor based agents resulted in longer PFS (HR 0.61, 95 % CI 0.43–0.88, *P* = 0.007, Fig. 5f) and OS (HR 0.75, 95 % CI 0.63–0.90, *P* = 0.002, Fig. 4f). Besides, due to the incidence of GC is region-specific and treatment approaches are different between eastern and western countries, so the patients enrolled in the study were also divided into two subgroups: the Asian group and the non-Asian group. Angiogenesis inhibitors increased PFS in both Asian patients (HR 0.62, 95 % CI 0.42–0.93, *P* = 0.02, Additional file 2: Figure S1C) and non-Asian patients (HR 0.61, 95 % CI 0.53–0.69, *P* < 0.00001, Additional file 2: Figure S1D), but only improved OS (HR 0.82, 95 % CI 0.70–0.95, *P* < 0.007, Additional file 2: Figure S1C) in non-Asian patients.

### Overall response rate (ORR) and disease control rate (DCR)

**Fig. 6:**
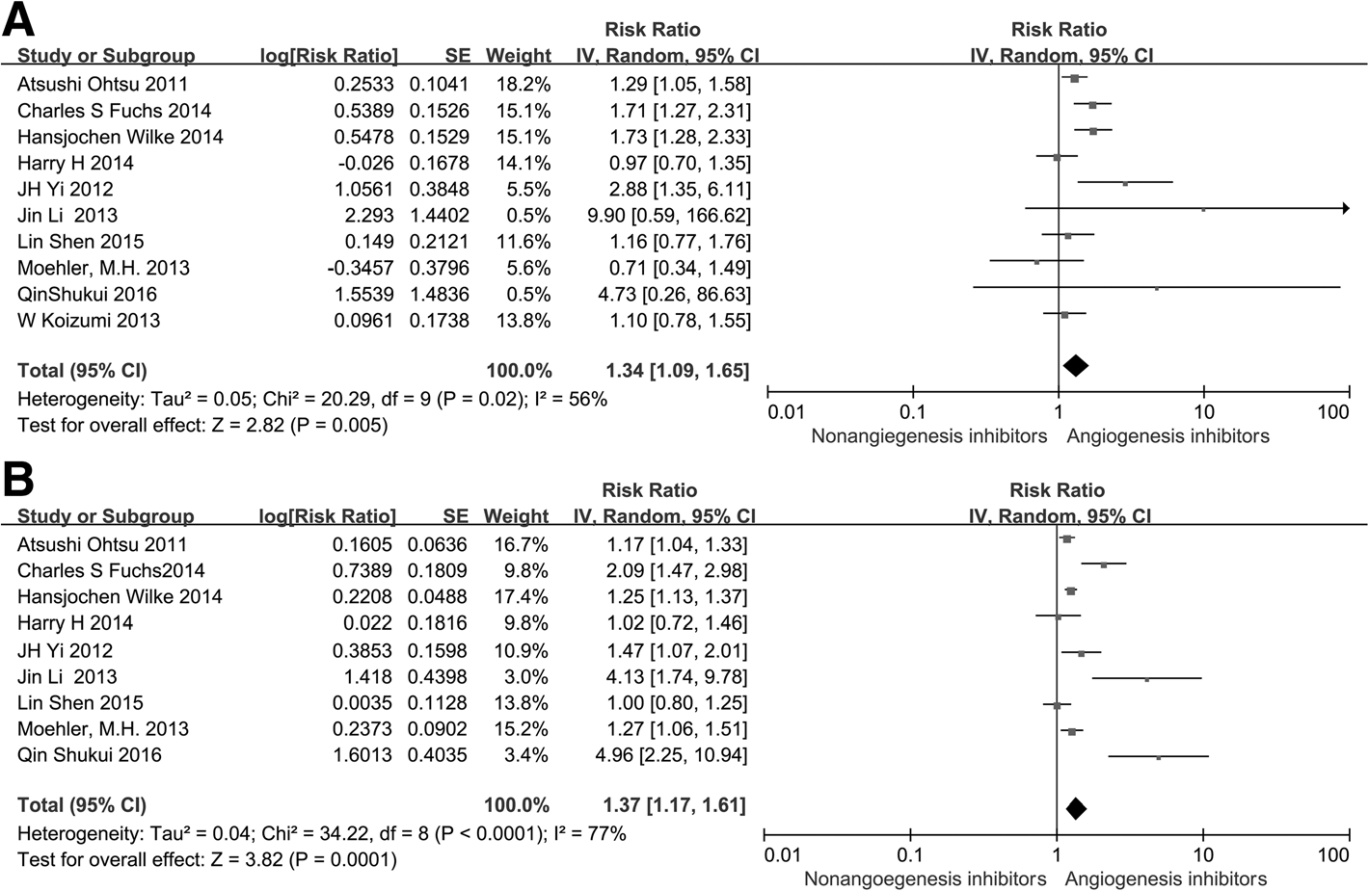
Forest plot and pooled RR and 95 % CI for DCR (**a**) and ORR (**b**): anti-angiogenesis therapy versus non-anti-angiogenesis therapy. The pooled RR for DCR and ORR showed that the patients receiving anti-angiogenesis therapy had superior DCR and ORR. *RR* risk ratios, *CI* confidence intervals, *ORR* overall response rate, *DCR* disease control rate

All ten trials reported ORR, and nine studies reported DCR. The DCR ranged from 0 to 85 %, and the ORR varied from 0 to 46 % in the angiogenesis inhibitors group. The pooled data showed that angiogenesis inhibitors resulted in superior ORR (HR 1.34, 95 % CI 1.09–1.65, *P* = 0.005, Fig. 6b) and a high DCR (HR 1.37, 95 % CI 1.17–1.61, *P* = 0.0001, Fig. 6a) compared with non-angiogenesis inhibitors.

**Fig. 7:**
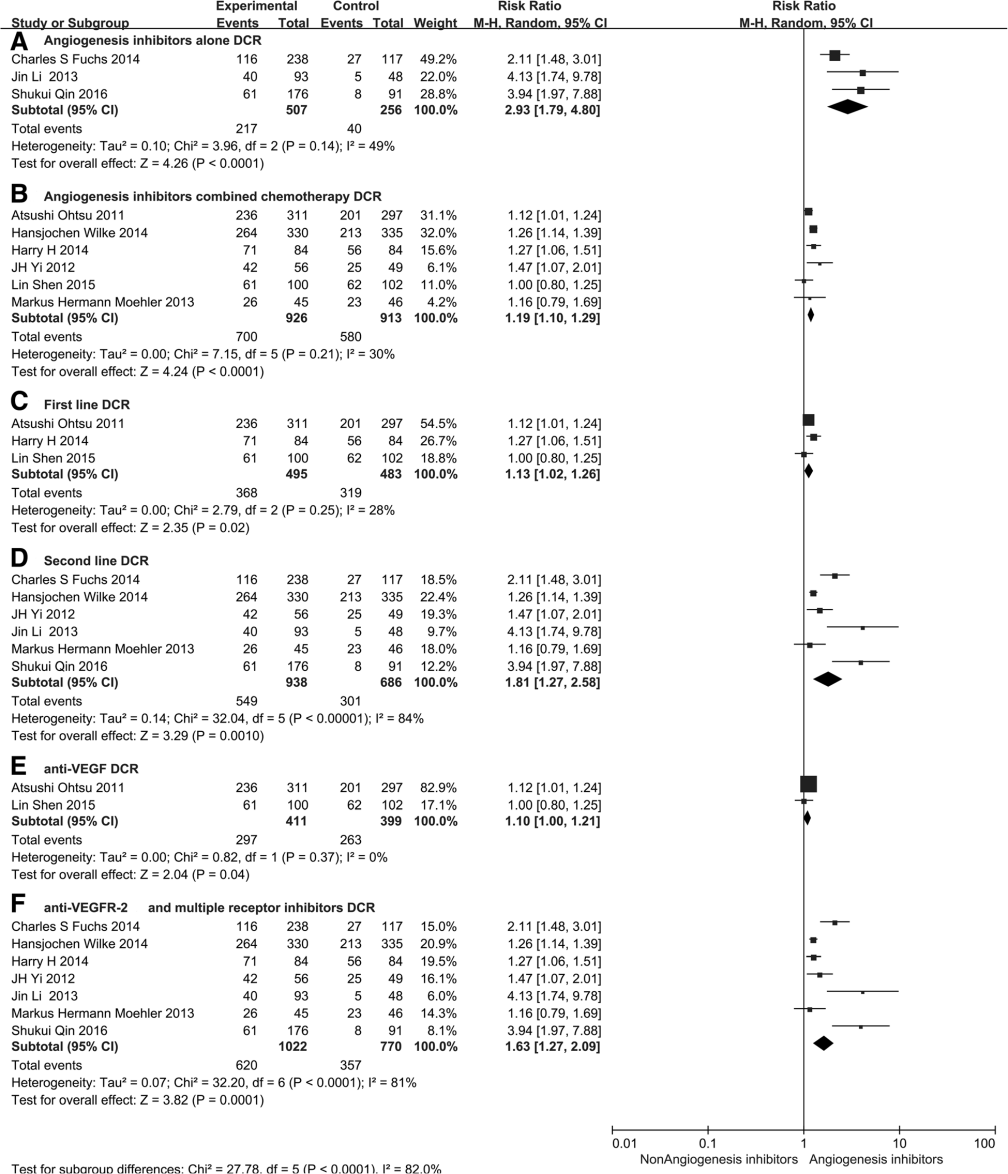
Forest plot and pooled RR and 95 % CI for subgroup DCR: anti-angiogenesis therapy versus non-anti-angiogenesis therapy. *RR* risk ratios, *CI* confidence intervals, *DCR* disease control rate. (**a**: DCR of subgroups of a﻿ngiogenesis inhibitors alone threapy; **b**: DCR of subgroups of angiogenesis inhibitors combined with chemotherapy threapy; **c**:﻿ DCR﻿ ﻿of subgroups of the first line﻿ ﻿thre﻿apy; **d**: DCR of subgroups of the second line threapy; **e**: DCR of subgroups of anti-VEGF threapy; **f**:﻿ DCR of subgroups of anti-VEGFR an﻿d multiple receptor inhibitors threapy﻿)﻿

**Fig. 8:**
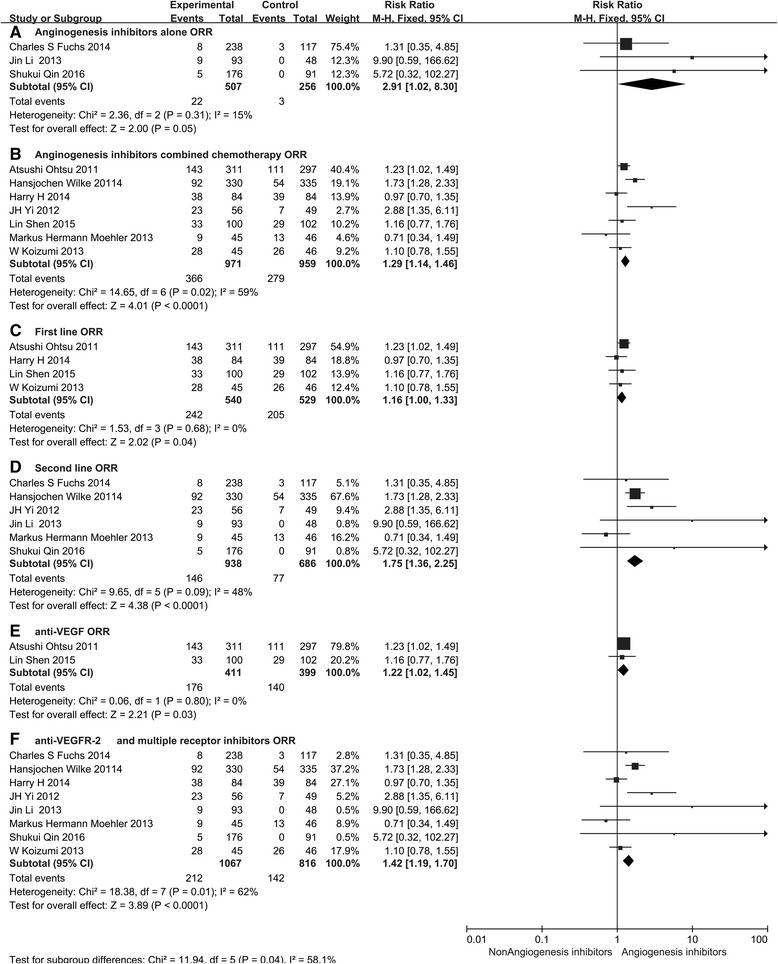
Forest plot and pooled RR and 95 % CI for subgroup ORR: anti-angiogenesis therapy versus non-anti-angiogenesis therapy. *RR* risk ratios, *CI* confidence intervals, *ORR* overall response rate. (**a**﻿: ORR of subgroups of a﻿ngiogenesis inhibitors alone threapy; **b**: ORR of subgroups of angiogenesis inhibitors combined with chemotherapy threapy; **c**:﻿ ORR of subgroups of the first line﻿ ﻿thre﻿apy; **d**: ORR of subgroups of the second line threapy; **e**: ﻿ORR of subgroups of anti-VEGF threapy; **f**: ORR of subgroups﻿ ﻿of anti-﻿VEGFR ﻿an﻿d multiple receptor inhibitors threapy﻿﻿)﻿

Subgroup analysis of medication administration indicated that both angiogenesis inhibitors alone and angiogenesis inhibitors combined with chemotherapy led to a statistically significant improvement in DCR (RR 2.93, 95 % CI 1.79–4.80, *P* < 0.0001, Fig. 7a and RR 1.19, 95 % CI 1.10–1.29, *P* < 0.0001, Fig. 7b). However, we only found an improvement in ORR (RR 1.29, 95 % CI 1.14–146, *P* < 0.0001, Fig. 8B) for patients treated with angiogenesis inhibitors combined with chemotherapy. In addition, angiogenesis inhibitors increased DCR and ORR in both the first-line (RR 1.13, 95 % CI 1.02–1.26, *P* = 0.02, Fig. 7c and RR 1.16, 95 % CI 1.00–1.33, *P* = 0.04, Fig. 8d) and ≥ the second-line therapy (RR 1.81, 95 % CI 1.27–2.58, *P* = 0.001, Fig. 7d and RR 1.75, 95 % CI 1.36–2.25, *P* < 0.0001, Fig. 8d). When stratified by drug class, improvement in ORR and DCR were observed with both anti-VEGF-based drugs (RR 1.22, 95 % CI 1.02–1.45, *P* = 0.03, Fig. 8e and RR 1.10, 95 % CI 1.00–1.21, *P* = 0.04, Fig. 7e) and anti-VEGFR-2-based and multiple receptor tyrosine kinase inhibitor drugs (RR 1.42, 95 % CI 1.19–1.70, *P* < 0.0001, Fig. 8f and RR 1.63, 95 % CI 1.27–2.09, *P* = 0.0001, Fig. 7f).

### Safety

The toxicity reported in the included studies, summarized according to the National Cancer Institute Common Toxicity Criteria is shown in Table 2 (only grade ≥3 toxicities are presented). In general, grade ≥3 adverse events were more frequent in patients treated with angiogenesis inhibitors and included hand-foot syndrome, hemorrhage, hypertension, proteinuria, and GI perforation for anti-angiogenic-induced events, and neutropenia, leukopenia, and fatigue for chemotherapy-induced events. In addition, hand-foot syndrome (RR 2.17, 95 % CI 1.48–4.97, *P* = 0.001, Table 2), diarrhea (RR 1.66, 95 % CI 1.11–2.50, *P* = 0.01, Table 2), and GI perforation (RR 4.13, 95 % CI 1.14–15.05, *P* = 0.03, Table 2) were significantly increased in patients treated with angiogenesis inhibitors. However, anemia (RR 0.73, 95 % CI 0.54–0.98, *P* = 0.03, Table 2) was less frequent in the angiogenesis inhibitor group. The RRs of grade ≥3 adverse events are summarized in Table 2.

**Table 2:** RR of grade ≥3 adverse events in patients with advanced gastric cancer treated with angiogenesis inhibitors

Grade ≥3 adverse events	No. of trials	Events/total		RR (95 % CI)	*P* value	Analysis model
		Treatment group	Control group			
Decreased appetite	5	55/1095	60/973	0.86 (0.61, 1.22)	0.39	Fixed
Vomiting	5	62/1051	67/975	0.94 (0.48, 1.82)	0.84	Random
Anemia	8	118/1419	125/1161	0.91 (0.55, 1.50)	0.7	Random
Hypertension	6	105/1318	22/1066	2.85 (0.87–9.30)	0.08	Random
Hemorrhage	4	27/898	31/698	0.65 (0.39–1.09)	0.11	Fixed
Thromboembolic events	4	42/778	56/598	0.68 (0.46, 1.00)	0.05	Fixed
Proteinuria	5	147/1218	141/529	1.07 (0.76–1.34)	0.94	Fixed
GI perforation	3	12/985	2/496	4.1 (1.14–15.05)	0.03	Fixed
Neuropathy	3	30/318	27/312	1.11 (0.68–1.82)	0.67	Fixed
Diarrhea	7	60/1135	35/1044	1.66 (1.11, 2.50)	0.01	Fixed
Nausea	6	45/996	58/952	0.77 (0.53, 1.11)	0.16	Fixed
Fatigue	7	79/904	55/627	0.91 (0.44, 1.88)	0.75	Random
Leukopenia	6	83/797	45/665	1.23 (0.61, 2.48)	0.58	Random
Thrombocytopenia	5	33/797	16/665	1.74 (0.96, 3.12)	0.07	Fixed
Neutropenia	7	217/842	118/711	1.53 (0.99, 2.37)	0.06	Random
Hand-foot syndrome	4	51/711	14/569	2.83 (1.57, 5.11)	0.0005	Fixed
Electrolyte disturbance	3	23/662	26/574	0.8 (0.32–1.99)	0.63	Fixed
Pain	6	42/694	31/676	1.06 (0.30, 3.79)	0.93	Random
Liver damage	4	56/414	19.287	1.59 (1.01, 2.51)	0.04	Fixed

*RR* risk ratios, *GI* gastrointestinal

## Discussion

GC is one of the most common malignant tumors of the digestive tract worldwide. With the development of therapeutic strategies, the survival time of GC patients has significantly increased over the past 20 years. However, the prospects for the treatment of GC are not optimistic. Chemotherapy is currently the main treatment for advanced GC; however, there is no standard first-line chemotherapy regimen for advanced GC. Moreover, traditional chemotherapy has reached an efficacy plateau. Therefore, it is necessary to identify a more effective treatment for advanced GC.

In the last 10 years, the rapid development of molecular biology has provided new directions for the treatment of GC. Many studies on immunotherapy and anti-angiogenic therapy are underway [28]. For example, the results of KEYNOTE-012 and KEYNOTE-028 indicated that pembrolizumab, a type of anti-PD-1 antibody, may be a promising agent in pretreated and PD-L1-positive advanced GC. However, there is still a long way to go before patients can benefit from this treatment. Among the novel target treatment strategies, the most widely studied, the most extensive, and the most in-depth is the study of angiogenesis. The angiogenic pathway modulated by the VEGF family has been extensively studied in many tumors [29]. Angiogenesis contributes to the progression, invasion, and metastasis of malignancy and the importance of anti-angiogenesis therapy in inhibiting malignant tumor growth has been confirmed. Targeting the VEGF pathway in GC started to receive more attention when the results from phase III trials confirmed its efficacy in inducing superior survival outcome beyond standard therapy [9, 10, 13]. Ramucirumab, a monoclonal antibody, can selectively bind to VEGFR-2 and block the downstream effects of the VEGF pathway in angiogenesis. The REGARD [9] and RAINBOW [10] studies reported that advanced GC patients may benefit from treatment with ramucirumab (HR_OS_ 0.776, 95 % CI 0.603–0.998, *P* = 0.047, HR_OS_ 0.807, 95 % CI 0.678–0.962, *P* < 0.0001, respectively, and HR_PFS_ 0.483, 95 % CI 0.376–0.620, *P* < 0.0001, HR_PFS_ 0.635, 95 % CI 0.536–0.752, *P* < 0.0001, respectively). The survival benefits in the REGARD and RAINBOW studies led to the approval of ramucirumab by the FDA for the treatment of advanced GC. Apatinib, a small molecule oral TKI, can inhibit the intracellular function of VEGFR by blocking the receptors of tyrosine kinases expressed by endothelial cells. A phase II [11] and phase III [13] trial explored the effects of apatinib in patients with advanced GC and the results showed that the patients benefited from apatinib treatment. Bevacizumab, a monoclonal antibody, can bind VEGF-A ligand, thus inhibiting VEGF-mediated angiogenesis. The results of the AVAGAST [25] study showed that bevacizumab can improve ORR (HR 8.61, 95 % CI 0.6–16.6, *P* = 0.0315) and prolong PFS (HR, 0.80; 95 % CI 0.68–0.93, *P* = 0.0037), but there was no significant difference in OS (HR 0.87, 95 % CI 0.73–1.03, *P* = 0.1002) when compared with the placebo. In addition, the outcomes of the AVATAR [26] study were similar to those of the AVAGAST study.

Fontana et al. [30] summarized the clinical efficacy of bevacizumab and ramucirumab in advanced GC and discussed the results of clinical trials but paid little attention on other angiogenic inhibitors, such as apatinib and sunitinib. Aprile et al. [31] mainly focused on the current status of novel angiogenesis inhibitors in advanced GC, the underlying biology, their mechanism of action, and recent clinical trial results. They suggested that VEGFR-2 plays a key role in GC, and VEGFR-2 blockade may be associated with improved outcomes. Future efforts in translational research should aim to clarify which patients may benefit from the anti-angiogenic therapy. The aforementioned reviews extensively describe the major clinical results of the angiogenic inhibitors, their efficiency and disadvantages in advanced GC. However, they are all narrative reviews and may lead to a number of methodological flaws without a clear and objective methods section. Furthermore, there is still controversy regarding the effects of angiogenesis inhibitors on advanced GC. Hence, we performed this updated meta-analysis to provide valuable clues for the clinical application of angiogenesis inhibitors.

According to the current results, regimens containing angiogenesis inhibitors showed substantial improvements in OS (HR 0.80, 95 % CI 0.69–0.93, *P* = 0.004, Fig. 3a), PFS (HR 0.66, 95 % CI 0.51–0.86, *P* = 0.002, Fig. 3b), ORR (HR 1.34, 95 % CI 1.09–1.65, *P* = 0.005, Fig. 6b), and DCR (HR 1.37, 95 % CI 1.17–1.11, *P* = 0.0001, Fig. 6a) compared with regimens without angiogenesis inhibitors. Subgroup analyses showed that OS was significantly improved following treatment with angiogenesis inhibitor monotherapy (HR 0.61, 95 % CI 0.42–0.89, *P* = 0.01, Fig. 4a) or combined with chemotherapy (HR 0.88, 95 % CI 0.79–0.97, *P* = 0.01, Fig. 4b) when compared with placebo and chemotherapy alone. However, angiogenesis inhibitor monotherapy was unable to prolong PFS (HR 0.86, 95 % CI 0.71–1.03, *P* = 0.1, Fig. 5a) in patients with advanced GC. The reason may be the anti-VEGF-based drugs combined with chemotherapy can prolong PFS (HR 0.82, 95 % CI 0.71–0.93, *P* = 0.003), while anti-VEGFR-2 and multiple tyrosine kinase receptor inhibitors combined with chemotherapy failed to improve PFS (HR 0.92, 95 % CI 0.65–1.03, *P* = 0.63). With regard to the line of treatment, the efficacy of angiogenesis inhibitor therapy may be different in the first-line and ≥ the second-line setting. Significant PFS (HR 0.50, 95 % CI 0.34–0.73, *P* = 0.0004, Fig. 5d), OS (HR 0.71, 95 % CI 0.58–0.88, *P* = 0.002, Fig. 4d), ORR (RR 1.75, 95 % CI 1.36–2.25, *P* < 0.0001, Fig. 8d), and DCR (RR 1.81, 95 % CI 1.27–2.58, *P* = 0.001, Fig. 7d) benefits were observed in ≥ the second-line setting. However, there were ORR (RR 1.16, 95 % CI 1.00–1.33, *P* = 0.04, Fig. 8c) and DCR (RR 1.13, 95 % CI 1.02–1.26, *P* = 0.02, Fig. 7c) gains, but no OS (HR 0.92, 95 % CI 0.80–1.06, *P* = 0.23, Fig. 4c) and PFS (HR 0.87, 95 % CI 0.75–1.01, *P* = 0.08, Fig. 5c) benefits in the first-line setting. This may be due to the results of the most RCTs showed no significant survival benefits regarding OS and PFS, which included in this meta-analysis. With respect to the anti-angiogenic drug class, subgroup analysis showed that anti-VEGFR-2 and multiple tyrosine kinase receptor inhibitor treatment was more efficacious than anti-VEGF treatment in terms of OS, PFS, ORR, and DCR. One possible explanation is the differences in the targets of the angiogenesis inhibitors. Anti-VEGFR-2 and multiple tyrosine kinase receptor inhibitor drugs selectively bind to VEGFR-2, which plays a key role in the VEGF/VEGFR pathway. Anti-VEGF drugs only bind to VEGF-A and thus cannot block other members of the VEGF family binding to VEGFR-2. Another possible reason is related to the two clinical trials included in the anti-VEGF subgroup, which included patients treated with bevacizumab who had negative survival outcomes. Ramucirumab and apatinib were included in the VEGFR-2 and multiple tyrosine kinase receptor inhibitor group. The mechanistic advantage of ramucirumab, which binds to VEGFR-2 and has a long half-life, may be better than bevacizumab. In addition, the recent phase III study of apatinib [13], an oral small molecule VEGFR-2 TKI, in Chinese patients with advanced GC, demonstrated prolonged median OS in the apatinib arm of 195 versus 140 days in the placebo arm (HR 0.71, 95 % CI 0.54–0.94, *P* < 0.0001). The reason for these differences is a current challenge, and further studies may elucidate the pharmacological differences and possibly improve clinical outcome.

As the incidence of GC is region-specific, and treatment approaches are different between eastern and western countries, we divided the patients into two subgroups: the Asian group and the non-Asian group. The results show that angiogenesis inhibitors increased PFS in both Asian patients (HR 0.62, 95 % CI 0.42–0.93, *P* = 0.02, Additional file 2: Figure S1C) and non- Asian patients (HR 0.61, 95 % CI 0.53–0.69, *P* < 0.00001, Additional file 2: Figure S1D) but only improved OS in non-Asian patients (HR 0.82, 95 % CI 0.70–0.95, *P* < 0.007, Additional file 2: Figure S1B). Given that angiogenesis is a host mechanism, the observed differences between Asian patients and non-Asian patients may be attributed to the inherent differences in these ethnic populations. This should be confirmed in future clinical trials by focusing on the influence of racial/ethnic factors.

Another focus in the treatment of GC patients is safety and tolerability. Angiogenesis inhibitors have more adverse reactions, such as hemorrhage, hypertension, and proteinuria, and most are predictable and manageable [12]. However, hand-foot syndrome (RR 2.83, 95 % CI 1.57–5.11, *P* = 0.0005), diarrhea (RR 1.66, 95 % CI 1.11–2.50, *P* = 0.01), and GI perforation (RR 4.10, 95 % CI 1.14–15.05, *P* = 0.03) were significantly increased in patients treated with angiogenesis inhibitors. VEGF is also a vital factor in angiogenesis in normal tissues. Consequently, anti-angiogenic agents can destroy the network of capillaries in healthy tissues, and this is the major reason underlying the adverse reactions of these drugs. In this meta-analysis, the safety of angiogenesis inhibitors was similar to previous results for non-small cell lung carcinoma. Gastrointestinal perforation and diarrhea are due to damage to the blood flow in normal tissues by angiogenesis inhibitors and lead to intestinal ischemia and necrosis, resulting in GI perforation and diarrhea. Hand-foot syndrome, a type of skin toxicity, is a known adverse reaction of angiogenesis inhibitors, although the mechanism of this reaction is unknown, it may be associated with the effects of angiogenesis inhibitors on endothelial cells, resulting in vascular bed degradation. The hands and feet are rich in capillaries and capillary degradation is likely to lead to abnormal sensation and changes in the skin.

Although the toxicity profiles of biologics (bevacizumab, ramucirumab) and small molecule TKIs (afatinib, sunitinib, and TSU-68) overlap but are not the same, we conducted a subgroup analysis. This analysis showed that the biologics are more likely to lead to hypertension (RR 5.87, 95 % CI 3.34–10.34, *P* < 0.0001, Additional file 3: Table S4), neutropenia (RR 1.56, 95 % CI 1.27–1.93, *P* < 0.0001, Additional file 3: Table S4), diarrhea (RR 1.83, 95 % CI 1.14–2.94, *P* = 0.01, Additional file 3: Table S4), and gastrointestinal perforation (RR 4.14, 95 % CI 1.14–10.09, *P* = 0.03, Additional file 3: Table S4), while small molecule TKIs are more likely to lead to hand-foot syndrome (RR 7.70, 95 % CI 1.83–32.39, *P* = 0.005, Additional file 4: Table S3) and thrombocytopenia (RR 0.68, 95 % CI 0.46–1.00, *P* = 0.04, Additional file 4: Table S3).

Although angiogenesis inhibitors can improve OS and PFS and achieve a better response rate in advanced GC, the clinical effect is quite different in individuals due to heterogeneity of the tumor. It is unclear which patients benefit most from angiogenesis inhibitors. In an effort to limit the toxicity and cost of therapy, a large number of basic and clinical studies need to be conducted, in order to identify biomarkers which can be used to predict efficacy and choose the most suitable patients to reduce the blindness of clinical medication.

There are many limitations in this meta-analysis. Firstly, a small number of trials were included, and there were no subgroups related to tumor pathological staging or pathological types. Moreover, the subgroup analysis included in the literature was limited to one type of angiogenesis inhibitor, and the conclusions were limited. Secondly, the differences between statistical quality, follow-up period, courses, and race in patients receiving angiogenesis inhibitors resulted in heterogeneity. Thirdly, the angiogenesis inhibitors included mainly targeted VEGF and its receptor family, thus the conclusions do not cover all types of angiogenesis inhibitors. Finally, this is a trial-level meta-analysis based on studies and not on individual patient data. Confounding variables such as patient co-morbidities, extent of disease, and differences in other possible prognostic factors could not be incorporated into this analysis. Therefore, future research should focus on high-quality studies and clinical features in patients with comprehensive evaluation, thus resulting in more standardized research and more accurate conclusions.

## Conclusions

Anti-angiogenic treatment was better than non-anti-angiogenic treatment in terms of OS, PFS, ORR, and DCR in patients with advanced GC. Further studies are needed to explore the timing and potentially predictive biomarkers of angiogenesis inhibitors to improve the selection of patients and improve clinical benefit.

## Additional files

Additional file 1: Table S5. The major trails of angiogenesis inhibitors in use for gastric cancer. (DOC 95.0 kb)
Additional file 2: Figure S1. Forest plot and pooled HR and 95 % CI for Asian patients OS (A) non-Asian patients OS (B) and Asian patients PFS (C) and non-Asian patients PFS (D): anti-angiogenesis therapy versus non-anti-angiogenesis therapy. HR, hazard ratios; CI, confidence intervals; PFS, progression-free survival; OS, overall survival. (DOC 72800 kb)
Additional file 3: Table S4. RR of grade ≥3 adverse events in patients with advanced gastric cancer treated with biologics of angiogenesis inhibitors. (DOC 42.0 kb)
Additional file 4: Table S3. RR of grade ≥3 adverse events in patients with advanced gastric cancer treated with TKI of angiogenesis inhibitors. (DOC 40.5 kb)
