# Supplementary material for: Efficacy and safety of angiogenesis inhibitors in advanced gastric cancer: a systematic review and meta-analysis
Source: J Hematol Oncol. 2016 Oct 18;9:111. doi: 10.1186/s13045-016-0340-8 (PMC5070169; doi:10.1186/s13045-016-0340-8)
Supplement: Additional file 1: Table S5. — The major trails of angiogenesis inhibitors in use for gastric cancer. (DOC 95.0 kb) [file 13045_2016_340_MOESM1_ESM.doc]

Table S5 The major trails of angiogenesis inhibitors in use for gastric cancer

| Trials |  | year | phase | Treatment | Patients  entrolled | Media age(yesrs) | mOS  (months) | mPFS  (months) |
| --- | --- | --- | --- | --- | --- | --- | --- | --- |
|  |  |  |  |  |  |  |  |  |
| Atsushi Ohtsu[1] |  | 2011 | 3 | CDP+Cap+placebo | 387 | 59 | 10.1 | 5.3 |
|  |  |  |  | CDP+Cap +Bev | 387 | 58 | 12.1 | 6.7 |
|  |  |  |  |  |  |  |  |  |
| Lin Shen[2] |  | 2015 | 3 | CDP+Cap +placebo | 102 | 55.5 | 10.5 | 6 |
|  |  |  |  | CDP+Cap +Bev | 100 | 54,.2 | 11.4 | 6.3 |
|  |  |  |  |  |  |  |  |  |
| EI-Rayes[3] |  | 2010 | 2 | TAX+Bev+LOHP | 38 | 57 | 11.1 | 6.6 |
|  |  |  |  |  |  |  |  |  |
| Hope E. Uronis[4] |  | 2013 | NR | Bev+LOPH+Cap | 37 | NR | 10.8 | 7.2 |
|  |  |  |  |  |  |  |  |  |
| [Shah MA[5]](http://www.ncbi.nlm.nih.gov/pubmed/?term=Shah MA%5BAuthor%5D&cauthor=true&cauthor_uid=17114652) |  | 2006 | 2 | Bev+CPT-11+DDP | 57 | 59 | 12.3 | NR |
|  |  |  |  |  |  |  |  |  |
| [Shah MA[6]](http://www.ncbi.nlm.nih.gov/pubmed/?term=Shah MA%5BAuthor%5D&cauthor=true&cauthor_uid=17114652) |  | 2011 | 2 | Bev+mDCF | 44 | 57 | 16.8 | 12 |
|  |  |  |  |  |  |  |  |  |
| Charles Fuchs[7] |  | 2014 | 3 | BSC+Placebo | 117 | 60 | 3.8 | 1.3 |
|  |  |  |  | BSC+Ram | 238 | 60 | 5.2 | 2.1 |
|  |  |  |  |  |  |  |  |  |
| Wilke H[8] |  | 2014 | 3 | PTX+Placebo | 335 | NR | 7.4 | 2.9 |
|  |  |  |  | PTX+Ram | 330 | NR | 9.6 | 4.4 |
|  |  |  |  |  |  |  |  |  |
| Yoon HH[9] |  | 2014 | 2 | Ram+mFOLFOX6 | 84 | NR | 11.7 | 6.4 |
|  |  |  |  | placebo+mFOLFOX6 | 84 | NR | 11.5 | 6.7 |
|  |  |  |  |  |  |  |  |  |
| RAINFALL[10] |  | 2016 | 3 | Cap+DDP+Ram |  | ongoing |  |  |
|  |  |  |  | Cap+DDP+Placebo |  |  |  |  |
|  |  |  |  |  |  |  |  |  |
| Jin Li[11] |  | 2013 | 2 | Apatinib 850mg | 47 | 55 | 4.83 | 3.67 |
|  |  |  |  | Apatinib 425mg | 46 | 53 | 4.27 | 3.2 |
|  |  |  |  | placebo | 48 | 54 | 2.5 | 1.4 |
|  |  |  |  |  |  |  |  |  |
| Shukui Qin[12] |  | 2016 | 3 | Apatinib | 176 | 58 | 6.50 | 2.6 |
|  |  |  |  | placebo | 91 | 58 | 4.70 | 1.8 |
|  |  |  |  |  |  |  |  |  |
| [Koizumi](https://www.researchgate.net/profile/Wasaburo_Koizumi) W[13] |  | 2013 | 2 | TSU-68+S-1/CDDP | 46 | 62 | 16.6 | 6.9 |
|  |  |  |  | S-1/CDDP | 47 | 63.5 | 15.45 | 7.1 |
|  |  |  |  |  |  |  |  |  |
| Moehler MH[14] |  | 2013 | 2 | sunitinib+FOLFIRI | 45 | NR | 10.50 | 3.60 |
|  |  |  |  | FOLFIRI | 46 | NR | 9.00 | 3.30 |
|  |  |  |  |  |  |  |  |  |
| JH Yi[15] |  | 2012 | 2 | sunitinib+docetaxel | 56 | 54 | 8.00 | 3.90(TTP) |
|  |  |  |  | docetaxel | 49 | 52 | 6.60 | 2.60(TTP) |
|  |  |  |  |  |  |  |  |  |
| M. Moehle[16] |  | 2011 | 2 | sunitinib | 52 | 59 | 5.81 | 1.28 |
|  |  |  |  |  |  |  |  |  |
| Y.J. Bang[17] |  | 2011 | 2 | sunitinib | 78 | 56 | 6.8 | 2.3 |
|  |  |  |  |  |  |  |  |  |
| Richard MM[18] |  | 2013 | 2 | Sorafenib+LOHP | 40 | 63 | 6.5 | 3 |
|  |  |  |  |  |  |  |  |  |
| W. Sun[19] |  | 2010 | 2 | Sorafenib+TXT+DDP | 44 | 58 | 13.6 | 5.8 |
|  |  |  |  |  |  |  |  |  |
| Wainberg, Z.A[20] |  | 2011 | 2 | Erlotinib+ mFOLFOX6 | 33 | 59 | 11 | 5.5 |
|  |  |  |  |  |  |  |  |  |
| R.K. Ramanathan[21] |  | 2014 | 2 | MK-2206 | 66 | 60.7 | 5 | 1.8 |
|  |  |  |  |  |  |  |  |  |
| M. Alsina[22], |  | 2011 | 1 | Telatinib+Cap+DDP | 48 | NR | Not Reached | 4.7 |
|  |  |  |  |  |  |  |  |  |
| [Eatock MM](http://www.ncbi.nlm.nih.gov/pubmed/?term=Eatock MM%5BAuthor%5D&cauthor=true&cauthor_uid=23108953)[23] |  | 2013 | 1 | Trebananib 10mg/kg+CX | 57 | NR | NR | 4.2 |
|  |  |  |  | Trebananib 3mg/kg+CX | 57 | NR | NR | 4.9 |
|  |  |  |  | placebo+CX | 57 | NR | NR | 5.2 |
|  |  |  |  |  |  |  |  |  |
| Breithaupt K[24] |  | 2013 | 2 | Pazopanib+FLOFOX | ongiong |  |  |  |
|  |  |  |  | Pazopanib |  |  |  |  |
|  |  |  |  |  |  |  |  |  |

Ram = Ramucirumab; BSC = best supportive care; mOS = median overall survival; mPFS = median progression-free survival; PTX = paclitaxel; Bev = bevacizumab; CDP/CDDP = cisplatin; Cap = capecitabine，TTP=time to progression, NR=Not Report, CPT-11=irinotecan；LOHP = Oxaliplatin; CX= cisplatin + capecitabine
