# Supplementary material for: Efficacy and safety of angiogenesis inhibitors in advanced gastric cancer: a systematic review and meta-analysis
Source: J Hematol Oncol. 2016 Oct 18;9:111. doi: 10.1186/s13045-016-0340-8 (PMC5070169; doi:10.1186/s13045-016-0340-8)
Supplement: Additional file 3: Table S4. — RR of grade ≥3 adverse events in patients with advanced gastric cancer treated with biologics of angiogenesis inhibitors. (DOC 42.0 kb) [file 13045_2016_340_MOESM3_ESM.doc]

Table S4. RR of grade ≥3 adverse events in patients with advanced gastric cancer treated with biologics of angiogenesis inhibitors.

| Grade≥3 Adverse events | No.of Trials | events/total | | RR  (95%CI) | P value | Analysis Model |
| --- | --- | --- | --- | --- | --- | --- |
|  |  | Treatment Group | Control Group |  |  |  |
| Fatigue | 3 | 77/625 | 23/536 | 1.06  (0.5-2.26) | 0.88 | Random |
| Vomiting | 4 | 65/985 | 66/927 | 1.00  (0.51, 1.98) | 1 | Random |
| Nausea | 3 | 42/813 | 53/812 | 0.79  (0.53, 1.16) | 0.23 | Fixed |
| Diarrhea | 3 | 46/813 | 25/816 | 1.83  (1.14, 2.94) | 0.01 | Fixed |
| Anemia | 4 | 76/1054 | 101/935 | 0.71  (0.40, 1.24) | 0.22 | Random |
| Hypertension | 4 | 88/1054 | 14/935 | 5.87  (3.34, 10.34) | ＜0.0001 | Fixed |
| Hemorrhage | 3 | 21/724 | 24/600 | 0.73  (0.40, 1.33) | 0.3 | Fixed |
| thromboembolic events | 3 | 40/724 | 54/600 | 0.68  (0.46, 1.00) | 0.05 | Fixed |
| Proteinura | 3 | 141/954 | 140/833 | 0.99  (0.82, 1.19) | 0.92 | Fixed |
| GI perforation | 3 | 12/724 | 2/600 | 4.14  (1.14, 15.09) | 0.03 | Fixed |
| Neutropenia | 3 | 170/514 | 110/521 | 1.56  (1.27, 1.93) | ＜0.0001 | Random |

RR, risk ratios; GI, Gastrointestinal
