# Supplementary material for: Efficacy and safety of angiogenesis inhibitors in advanced gastric cancer: a systematic review and meta-analysis
Source: J Hematol Oncol. 2016 Oct 18;9:111. doi: 10.1186/s13045-016-0340-8 (PMC5070169; doi:10.1186/s13045-016-0340-8)
Supplement: Additional file 4: Table S3. — RR of grade ≥3 adverse events in patients with advanced gastric cancer treated with TKI of angiogenesis inhibitors. (DOC 40.5 kb) [file 13045_2016_340_MOESM4_ESM.doc]

Table S3. RR of grade ≥3 adverse events in patients with advanced gastric cancer treated with TKI of angiogenesis inhibitors.

| Grade≥3 Adverse events | No.of Trials | events/total | | RR  (95%CI) | P value | Analysis Model |
| --- | --- | --- | --- | --- | --- | --- |
|  |  | Treatment Group | Control Group |  |  |  |
| Hypertension | 2 | 17/223 | 8/139 | 1.76  (0.07, 41.44) | 0.73 | Random |
| Proteinura | 2 | 6/269 | 1/139 | 2.27  (0.38, 13.58) | 0.37 | Random |
| Fatigue | 3 | 10/359 | 13/231 | 0.61  (0.26, 1.42) | 0.25 | Fixed |
| Nausea | 3 | 3/183 | 5/140 | 0.60  (0.18, 2.01) | 0.41 | Fixed |
| Diarrhea | 4 | 14/322 | 10/232 | 1.27  (0.57, 2.83) | 0.57 | Fixed |
| Anemia | 4 | 42/370 | 24/234 | 1.48  (0.76, 2.85) | 0.25 | Fixed |
| Leukopenia | 4 | 22/370 | 15/234 | 1.17  (0.63, 2.18) | 0.62 | Fixed |
| Neutropenia | 5 | 70/415 | 38/280 | 1.60  (0.89, 2.90) | 0.12 | Random |
| Hand-foot syndrome | 3 | 28/325 | 1/188 | 7.70  (1.83, 32.39) | 0.005 | Fixed |
| Thrombocytopenia | 4 | 24/370 | 8/234 | 0.68  (0.46, 1.00) | 0.04 | Fixed |

RR, risk ratios; GI, Gastrointestinal
